# Chemiluminescent 2-Coumaranones: Synthesis, Luminescence Mechanism, and Emerging Applications

**DOI:** 10.3390/molecules30071459

**Published:** 2025-03-25

**Authors:** Stefan Schramm, Tim Lippold, Isabelle Navizet

**Affiliations:** 1 Applied Organic Chemistry, University of Applied Sciences Dresden, Friedrich-List-Platz 1, 01069 Dresden, Germany; 2 Institute of Organic Chemistry, Department of Chemistry, University of Cologne, Greinstr. 4, 50939 Cologne, Germany; 3 Univ Gustave Eiffel, Univ Paris Est Creteil, CNRS, UMR 8208, MSME, F-77454 Marne-la-Vallée, France

**Keywords:** 2-coumaranone, chemiluminescence, alpha-peroxylactone, 1,2-dioxetanone, Tscherniac–Einhorn reaction

## Abstract

Recently, 2-Coumaranones have emerged as a highly promising class of chemiluminescent compounds, distinguished by their unique structural properties that facilitate efficient light emission. This review provides a comprehensive analysis of their synthesis, structural characteristics, and chemiluminescence mechanisms, integrating historical perspectives with the latest advancements in the field. Beyond their intrinsic photophysical and chemical properties, 2-coumaranones have demonstrated broad utility across bioanalytical and material sciences. Notable applications include enzyme-catalyzed chemiluminescence in aqueous systems, glucose and urease-triggered detection assays, and mechano-base-responsive luminescence for stress sensing. Additionally, recent developments in chemiluminescent protective groups and their incorporation into advanced functional materials underscore the versatility of these compounds. Despite significant progress, key challenges remain, particularly in optimizing quantum yield, emission properties, and solvent compatibility for practical applications. Future research should prioritize the development of highly tunable 2-coumaranone derivatives with enhanced spectral and kinetic properties, further expanding their potential in diagnostics, bioimaging, and mechanoluminescent sensing. By addressing these challenges, 2-coumaranones could pave the way for next-generation chemiluminescent technologies with unprecedented sensitivity and adaptability.

**Dedicated to PD. Dr. Dieter Weiß in the occasion of his retirement from active academic life**

This review is dedicated to Dieter Weiß, whose visionary guidance as mentor and infectious passion for luminescent molecules profoundly shaped the main author’s academic journey. It was his early encouragement that set the main author on the path to explore 2-coumaranones, igniting a lifelong fascination with the chemistry of light and luminescent materials. Over the years, Weiß ‘s work at the Friedrich-Schiller University of Jena not only advanced practical organic chemistry but also inspired generations of students with unforgettable demonstrations that showcased the beauty and wonder of luminescent phenomena. Weiß’s ability to captivate audiences—whether through innovative experiments or the iconic presence of his “Dederon” Bag—has left an indelible mark on colleagues, students, and the wider scientific community alike. As he retires from active academic life, we celebrate not only his remarkable contributions as a researcher but also his enduring legacy as an educator and mentor. His relentless curiosity and commitment to uncovering new luminescent horizons will continue to inspire and illuminate the field for years to come.

## 1. Introduction

For millennia, humans have been fascinated by the glow of fireflies, bioluminescent beetles, jellyfish, and fungi. As early as 384—322 BCE, Aristotle described the luminescence of various organisms in his work *De Anima*, clearly distinguishing this “cold” light from the “hot” light produced by combustion [1,2]. Today, we recognize many forms of luminescence, including chemiluminescence, which refers to light emitted during a chemical reaction. The quantum chemical understanding of such reactions allows us to describe the processes involved at a fundamental level [3,4,5,6,7]. In a typical chemiluminescent reaction, a molecule (M) reacts to form a product (P), passing through a transition state (TS). During this process, the system undergoes a conical intersection, where the ground and excited states are energetically close, leading to the population of an excited state. This results in chemically excited molecules that can emit light when returning to their ground state, which is observed as chemiluminescence. When such reactions occur in biological organisms and are catalyzed by enzymes, they are referred to as bioluminescence [8,9,10,11].

**Figure 1:**
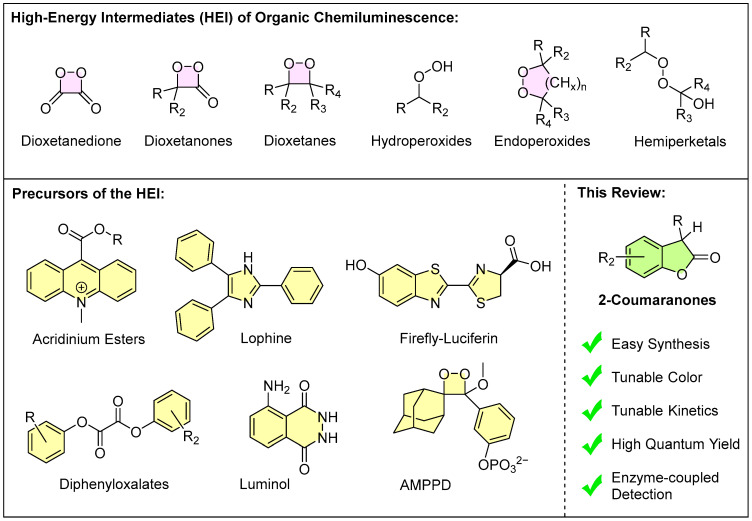
Overview of peroxide containing high-energy intermediates in organic chemiluminescence and important precursors of these molecules among which the scope of this review, the chemiluminescent 2-coumaranones, is highlighted.

There are numerous organic chemiluminescent systems, and all known systems share a common feature: they contain a high-energy peroxide intermediate, the decomposition of which leads to chemically excited states. Structures capable of chemiluminescence include dioxetanes, dioxetanones, endoperoxides, hydroperoxides, and hemiperketals, as well as precursors that form such structures during a chemical reaction. In bioluminescence, the chemiluminescent structure is typically a dioxetanone-like (alpha-peroxylactone) intermediate, as seen in firefly bioluminescence. (Figure 1) Chemiluminescent molecules have found widespread use in bioanalytical assays, such as luminol [12,13,14], AMPPD (a dioxetane analog) [15,16,17], and acridinium esters and derivatives [18,19,20,21]. However, there remains a need for further research to address challenges in efficiency (quantum yield) [22,23,24], kinetics (glow vs. flash emission) [25], and emission color [26,27,28], and to develop applications that surpass current methods.

This review aims to compile the current knowledge of chemiluminescent systems featuring the structural motif of 2-coumaranones (Figure 1). After providing a historical overview of the development of this research area, we will focus on their efficient synthesis, structure–property relationships, the mechanism of their chemiluminescence, and the potential applications derived from these insights. The goal is to consolidate the findings on this chemiluminescent system and to outline directions for future research.

## 2. Historical Development

Until recently, it has been believed that chemiluminescence of 2-coumaranones was first reported in a 1979 paper by G.J. Lofthouse, describing their accidental discovery during the synthesis of non-proteinogenic amino acids. The phenomenon was observed when 2-coumaranones were treated with bases like triethylamine in polar aprotic solvents [29]. However, earlier accounts of 2-coumaranone chemiluminescence can be traced back further. Lofthouse’s 1977 PhD thesis at the University of Salford, UK reveals that the phenomenon was first observed by B. Tuck at Ciba-Geigy (UK) Ltd. during amino acid synthesis, as indicated through personal communication. Additionally, R.A. Whittaker’s 1974 thesis from the University of Salford, UK includes related studies, confirming that 2-coumaranone chemiluminescence likely originated around 1970 as a serendipitous discovery during synthetic procedures involving phenylglycine derivatives [30,31].

Lofthouse’s early work included a variety of synthetic routes to 2-coumaranones, many involving lengthy, multi-step procedures with low overall yields and a narrow substrate scope [31]. One notable route he reported involved the alpha-hydroxy-N-alkoxy-glycine-mediated alpha-amidoalkylation, a reaction resembling electrophilic aromatic substitution. While Lofthouse cited D. Ben-Ishai’s 1975 studies as the origin of this reaction [32,33], its roots can be traced back to early 20th-century work. The German chemist J. Tscherniac first described the condensation of N-hydroxymethylphthalimide with aromatic compounds in a 1901 patent and a subsequent 1902 publication [34,35]. This methodology was expanded in 1905 by A. Einhorn, who developed an acid-promoted aromatic alkylation using N-hydroxymethylamides or imides, leading to the now widely recognized Tscherniac–Einhorn reaction [36,37].

After Lofthouse’s 1979 publication, the topic faded into obscurity until rediscovered by the Matthies research group at the University of Hamburg, Germany. Focusing on C-aryl glycines for pharmaceutical applications, Matthies’s team explored amidoalkylation reactions and their cytostatic properties, which structurally resemble 2-coumaranones [38,39]. This work led to a series of related PhD theses, notably A. Vogt’s exploration of inverse alpha-amidoalkylation in 1989 and B. Matuszczak’s detailed studies on 2-coumaranone synthesis, chemiluminescence mechanisms, and potential applications in 1991 [40,41]. Matuszczak later published a series of four papers between 1996 and 1998, extending the substrate scope, developing analytical assays, and introducing derivatives with N-acetyl side chains [42,43,44,45,46].

The topic remained dormant until rediscovered, serendipitously, in 2011 by Stefan Schramm during his studies in the laboratory of Dr. Dieter Weiß and Prof. Dr. Rainer Beckert at Friedrich-Schiller University of Jena, Germany. This rediscovery initiated a wave of publications exploring 2-coumaranones and their mechanisms and applications. Schramm’s collaborations with international researchers elucidated the involvement of 1,2-dioxetanone as the high-energy intermediate (HEI), clarified the chemiluminescence mechanism and provided the first reports of chemiluminescence quantum yields, which reached up to ~8%, significantly surpassing common analytical system like luminol [47,48,49,50,51,52].

This prompted investigations by other researchers into applications, including mechano-base-induced chemiluminescence by Sijbesma et al. in 2016 [53], enzyme-probing urea derivatives by Krieg et al. in 2019 [54], and modern synthetic approaches summarized by Baranov in 2020 [55]. Recently, the Griesbeck group developed 2-coumaranone derivatives as chemiluminescent protective groups, with Lippold contributing through his 2023 PhD thesis [56].

Through these efforts, 2-coumaranones have transitioned from an obscure chemiluminescent curiosity to a promising system with diverse synthetic, mechanistic, and application-focused advancements.

## 3. Synthesis

**Figure 2:**
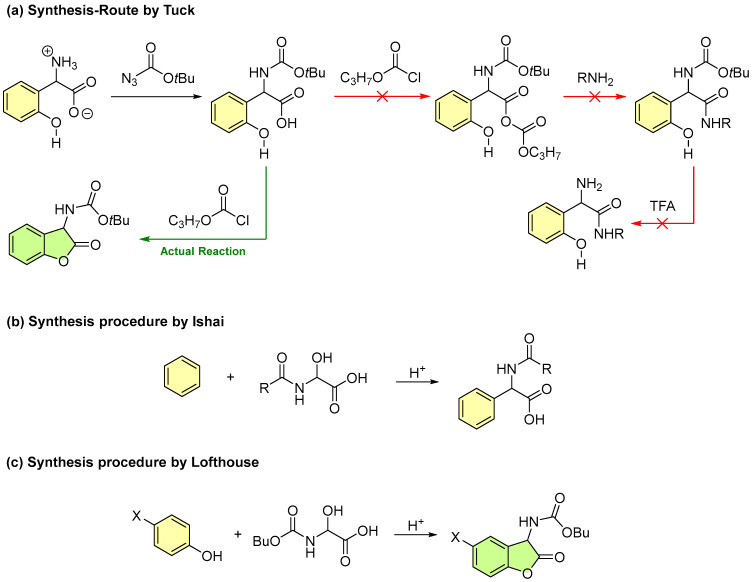
(**a**) Planned synthesis route by Tuck. The path with the green arrow represents the actual reaction that occurred; The one with the red arrow were not observed; (**b**) Original synthesis protocol by Ben-Ishai [33]; (**c**) Adaptation by Lofthouse [29,31].

The first documented synthesis of a 2-coumaranone was accidentally performed by Tuck attempting to prepare 2-amino-2-(2-hydroxyphenyl)acetamide derivatives (Figure 2a) [30]. Although the synthetic route was successfully established using phenylglycine as the starting material, the additional *ortho*-hydroxy group reacted further with an activated propyl carbonic anhydride intermediate, leading to the elimination of propyl carbonate and the formation of 2-coumaranone.

Building on Tuck’s findings, Lofthouse focused on synthesizing amino acids as a key precursor for producing benzofuran-2(3*H*)-ones with unsubstituted aromatic rings. However, the multi-step synthesis, based on the Bucherer–Bergs reaction, is cumbersome and time-consuming. To address these challenges, Lofthouse optimized the process using a protocol from Ben-Ishai, who employed the Tscherniac–Einhorn reaction with aromatic compounds and glyoxylic acid-amide adducts. By reacting *para*-substituted phenols (to prevent *ortho*-substitution) with α-hydroxy-*N*-(n-butoxycarbonyl)glycine in acetic acid containing 10% sulfuric acid, Lofthouse successfully synthesized a series of novel 2-coumaranones (Figure 2b,c) [29,31].

Compared to the original method by Tuck, which results in the formation of the 2-coumaranone in only very small yields, the usage of the Tscherniac–Einhorn reaction has been reported to achieve yields up to ~91% [50].

Key factors influencing the reaction include time and temperature. While longer reaction times often enhance yield and purity, higher temperatures can reduce non-condensed products but may also lead to unwanted by-products that are difficult to work up. To improve ring closure, acetic anhydride proved to be an effective reagent. Additionally, switching the solvent from sulfuric acid/acetic acid mixtures to trifluoroacetic acid (TFA) could also often improve the overall yield [56,57].

The same synthesis path has been used by all authors after Lofthouse; nevertheless, most considerable improvements have been made by translating the multi-step synthesis procedure into an efficient “One-Pot” reaction named the Tscherniac–Einhorn 3-component reaction [49,50].

**Figure 3:**
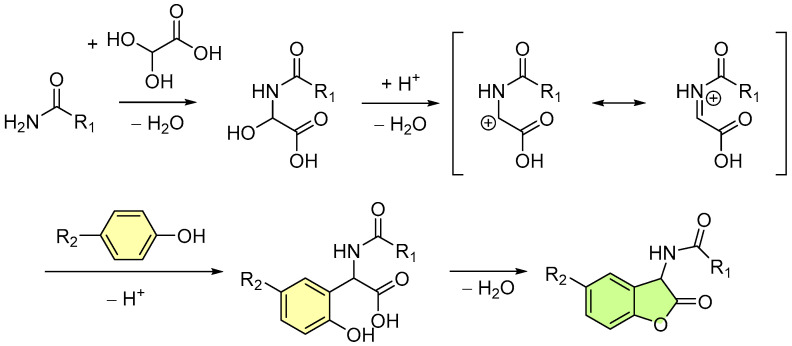
Mechanism of the modern “One-Pot” Tscherniac–Einhorn 3-component reaction forming a 2-coumaranone.

In regard to the mechanism of the Tscherniac–Einhorn 3-component reaction, an amide or carbamate first condenses with glyoxylic acid monohydrate. Acid catalysis then facilitates the elimination of a second water molecule, generating an iminium ion. The *para*-substituted phenol compound undergoes electrophilic aromatic substitution in the *ortho* position by the cationic intermediate thus formed. Finally, ring closure completes the synthesis, producing the target 2-coumaranone derivative as a lactone (Figure 3) [50].

To date, the reaction of phenols with glyoxylic acid derivatives remains a facile and efficient method and is widely recognized as the preferred approach for synthesizing 2-coumaranones. Beyond their potent chemiluminescent properties, benzofuranones have garnered increasing research interest due to their natural occurrence, which makes them promising candidates for potential pharmacological applications. Consequently, a broader range of synthetic procedures has emerged, enabling the targeted synthesis of benzofuranones with specific substitutions on the aromatic ring and/or the α-carbon of the lactone moiety, thereby expanding the catalogue of available derivatives.

## 4. Structure and Activity Relationships

**Figure 4:**
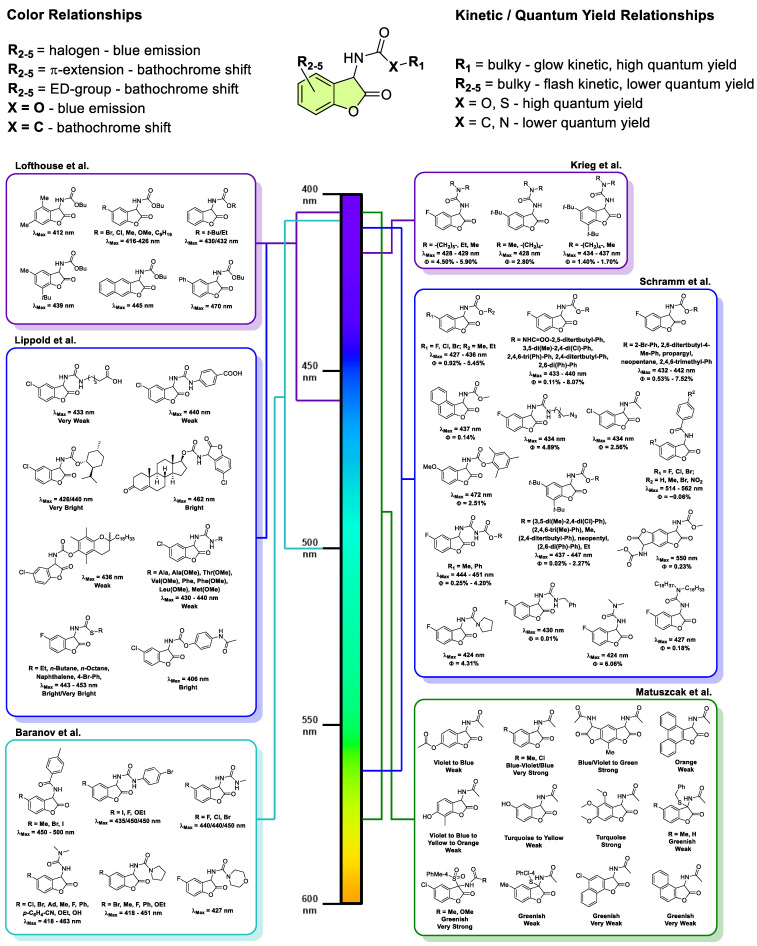
Overview of over 110 chemiluminescent 2-coumaranones reported in the literature to date (grouped according to the work of Lofthouse [29,31,58], Matuszcak [41,42,43,45,46], Schramm [47,48,49,50,51,52], Krieg [54], Lippold [56,57], and Baranov [59]). Here, 2-Coumaranones with the same basic scaffold were summarized and ordered either according to their emitting wavelength or quantum yield (lowest to highest value), if it has been determined.

So far, over 110 distinct molecules from the class of chemiluminescent 2-coumaranones have been reported in the literature. These derivatives display remarkable diversity in their chemiluminescence efficiency, kinetics, and emission color (Figure 4). The variability in these properties underscores the delicate interplay between molecular structure and photophysical/photochemical behavior. Precise spectroscopic values can be found in the Supplementary Information (Table S1, Figure S1).

**Figure 5:**
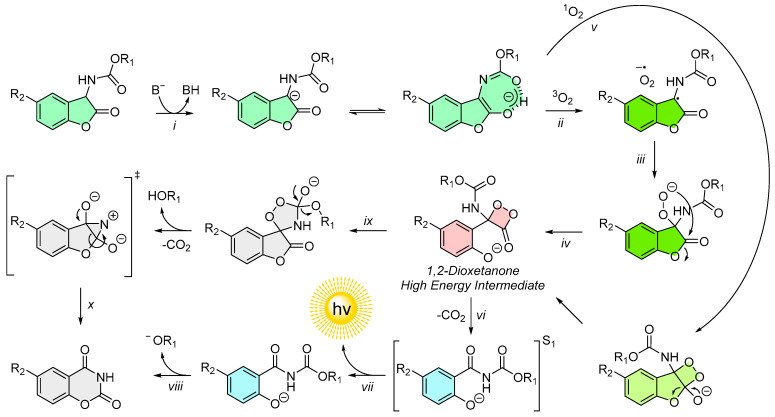
Experimentally and theoretically elucidated mechanism of 2-coumaranone chemiluminescence.

Most 2-coumaranone derivatives emit deep blue light (420–450 nm). However, structural modifications—particularly those that extend the π-conjugated system—can induce significant bathochromic shifts, resulting in green to orange emissions. Detailed investigations by Schramm et al. have revealed that the chemiluminescent light emitter is a deprotonated salicylamide-like structure (Figure 5) [50,51,52].

The intense blue photoluminescence of this emitter is a result deprotonation that generates a phenolate group with enhanced electron density, which acts as a strong electron donor. The close *ortho* positioning of this phenolate to an electron withdrawing amide/imide moiety facilitates effective orbital overlap. The highest occupied molecular orbital (HOMO) is primarily localized on the donor, while the lowest unoccupied molecular orbital (LUMO) is centered on the acceptor. This configuration promotes a robust intramolecular charge transfer (ICT) upon excitation, leading to a pronounced change in the dipole moment that is characteristic of ICT systems. The precise nature of this ICT has been experimentally and computationally investigated by Schram et al. [51,52].

Furthermore, the efficiency of the ICT process is highly sensitive to both molecular conformation and the extent of π-delocalization. Strategic substitution can enhance these effects: for example, the incorporation of electron donating groups, such as methoxy in the para position relative to the phenolate oxygen, increases the electron donating capacity of the donor, thereby intensifying the charge transfer and shifting the emission to a teal color (~475 nm). In addition, modifications to the central aromatic system serve as another powerful tool for tuning emission color. Matuszcak demonstrated that replacing a benzene core with a naphthalene framework extends charge delocalization and shifts the emission from blue to green, while further extension to a phenanthrene system narrows the HOMO–LUMO gap even more, yielding orange emission in the 560–580 nm range [45].

In addition to modifications of the central aromatic ring, extending the π-conjugation along the upper amide/carbamate sidechain similarly results in a bathochromic emission shift, as shown by Schramm et al. [47]. This observation suggests that combining both strategies—modifying the central aromatic system and the sidechain conjugation—could potentially enable emission in the deep red or near-infrared (NIR) region, a prospect of significant interest for deep tissue bioanalytical imaging applications.

Kinetic studies have further illuminated the structure–activity relationships in these systems. Schramm’s investigations revealed that sterically demanding carbamate sidechains tend to produce slow, glow-type chemiluminescence kinetics, while the incorporation of bulky alkyl groups, such as *t*-butyl substituents on the benzene core, facilitates fast, flash-type kinetics [50,51,52]. These findings highlight the sensitivity of the chemiluminescence process to subtle steric modifications.

Quantitative assessments of chemiluminescence quantum yields, although limited to a subset of reported 2-coumaranones, have shown striking differences among derivatives. Notably, molecules bearing carbamate and sterically hindered carbamate sidechains have exhibited quantum yields as high as ~8%—approximately an eightfold improvement over the benchmark luminol system [50]. However, it is important to note that the determination of chemiluminescence quantum yields is subject to significant experimental uncertainty, meaning that only substantial variations in yield are confidently interpretable.

The superior quantum yields of these sterically encumbered derivatives can be rationalized by the suppression of competing dark reaction pathways. In these systems, the bulky carbamate group impedes nucleophilic attack at the carbamate carbon, thereby reducing non-radiative decay routes (Figure 5). In contrast, replacing the carbamate group with an amide or urea typically leads to a drastic decrease in quantum yield. The higher reactivity associated with urea functionalities appears to promote alternative dark reaction pathways, as documented by Lippold et al., ultimately detracting from the chemiluminescence performance [56,57].

## 5. Mechanism of Chemiluminescence

The first ideas regarding the mechanism of chemiluminescence in 2-coumaranones originated from the work of Lofthouse. His research highlighted the dependence of the reaction on molecular oxygen and proposed the formation of a high-energy 1,2-dioxetanone intermediate. This hypothesis drew parallels with mechanisms observed in bioluminescent systems. The isolation of a dimer species provided further evidence for radical intermediates playing a role in the chemiluminescence pathway [29,31].

Further investigations by Matuszczak provided evidence for the formation of 2-coumaranone DBU salts. These studies suggested a critical deprotonation step at the α-position of the carboxy group in the lactone ring, initiating the chemiluminescence process [41,42,43,45,46].

The mechanism was subsequently explored in detail by Schramm et al., starting in 2013. Their comprehensive approach combined the chemical isolation of final products and intermediates with spectroscopic techniques, including photophysical, photochemical, and kinetic investigations. These experiments were complemented by quantum chemical calculations.

The investigations revealed several key mechanistic insights. Initially, deprotonation of 2-coumaranone generates a resonance-stabilized anion, which follows one of two major reaction pathways. In the first pathway, the anion undergoes single-electron transfer with triplet oxygen to produce a peroxyanion intermediate. In the alternative pathway, the anion reacts directly with singlet oxygen, leading to the formation of a 1,2-dioxetanone [51,52].

The peroxyanion from the triplet oxygen pathway subsequently cyclizes also into a 1,2-dioxetanone, the high-energy intermediate (HEI) whose decomposition represents the rate-limiting step [48]. During its breakdown, carbon dioxide is released, and an excited-state chemiluminescent emitter is generated through a conical intersection, subsequently relaxing to the ground state with the emission of light [51].

The final product of this reaction is a cyclic compound belonging to the carsalam class (1,3-benzoxazine-2,4-dione). However, spectroscopic analyses indicated that this product neither fluoresces nor matches the expected spectral properties of the chemiluminescent emitter. Instead, the true emitter was determined to be a deprotonated N-acyl-salicylamide derivative [50,51].

Additionally, computational studies uncovered a “dark” reaction pathway, via the formation of spirodioxazolidinone intermediates. This non-luminescent pathway results in the same carsalam class final reaction products as the light-emitting mechanism but without the generation of light [52].

Every critical step in the chemiluminescence reaction mechanism was followed using quantum chemical intrinsic reaction coordinate (IRC) calculations at the density functional theory level (B3LYP/6-31+G(d,p)) in polarizable continuum model (PCM) of MeCN solvent, with transition states identified and characterized. Final reaction products and light emitter species, as well as the electronic transitions at these geometries, were calculated both at time dependent TD-DFT and the post-Hartree–Fock complete active space self-consistent field completed by dynamic correlation CASSCF/CASPT2 level of theory (using ANO-RCC-VTZP basis set in a (14,12) active space) and validated by synthesis and spectroscopic analysis [50,51,52].

The commonly accepted mechanism for the chemiluminescence of 2-coumaranones is illustrated below (Figure 5):

(1) The 2-coumaranone is deprotonated by a base (*i*), forming a resonance-stabilized anion.
(2) The anion undergoes single-electron transfer with triplet oxygen (*ii*), producing a peroxyanion (*iii*), which cyclizes into a 1,2-dioxetanone (*iv*). Alternatively, it can directly react with singlet oxygen (*v*) to form the same 1,2-dioxetanone.
(3) The dioxetanone decomposes via a chemically induced electron exchange luminescence (CIEEL) mechanism, releasing CO_2_ and generating excited-state emitter molecules (*vi*).
(4) Upon relaxation to the ground state, light is emitted (*vii*) and, under basic conditions, the reaction completes with the formation of a carsalam product (*viii*).
(5) Alternative pathways, such as the formation and decomposition of spirodiox-azolidinone intermediates (*ix*), lead to the same final product via “dark” reactions (*x*).

**Figure 6:**
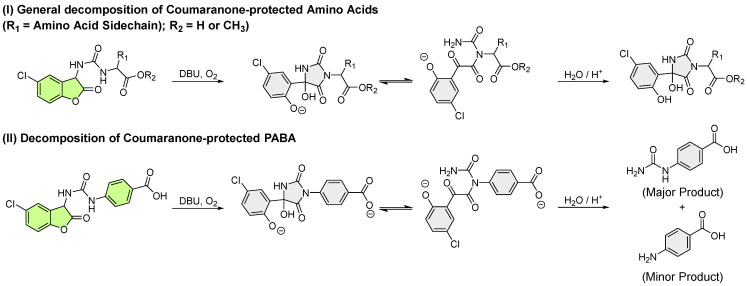
Dark reaction pathways of urea containing 2-coumaranones: (**I**) Decomposition reaction of 2-coumaranone-protected amino acids and esters. The fluorescent species is assumed to be open chain compound which could be detected after the oxidation process; (**II**) Decomposition of 2-coumaranone-protected PABA. In an alkaline solution an equilibrium between the hydantoin and open-chain derivative could be determined. Acidic workup yielded urea-protected PABA and fully deprotected PABA.

This chemiluminescence mechanism is highly influenced by the nature of the side chain attached to the molecule (e.g., OR_1_ in Figure 5). For derivatives with carbamate or thiolcarbamate side chains, the described mechanism holds true in its entirety. In the case of ureas, a study by Griesbeck et al. has shown that while the fundamental steps remain consistent, the reactive nature of ureas leads to variations in the dark reaction pathways, which in turn negatively affect the chemiluminescence properties (Figure 6) [56,57]. These dark reaction pathways will be closer discussed in the chapter on chemiluminescent protection groups (CLPGs).

For derivatives with amide side chains, the final cyclization to a carsalam structure does not occur due to the greater hydrolytic stability of amides compared to carbamates [47,49].

## 6. Applications of 2-Coumaranone Chemiluminescence

**Figure 7:**
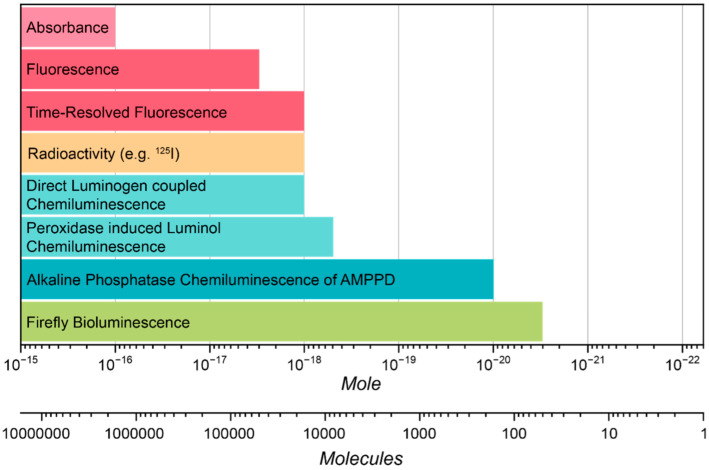
A comparison of limit of detection of immunoassays using different detection principle. adapted from the literature [58,63].

Chemiluminescence has been widely applied across various fields, demonstrating its utility in areas ranging from bioanalytics to material science [60,61,62]. Among these, bioanalytical applications stand out as the most prominent. Chemiluminescence-based assays often aim to replace traditional absorbance or fluorescence-based methods due to their unique advantages. Unlike absorbance and fluorescence systems, chemiluminescent assays do not require external light sources for excitation. This eliminates issues such as scattering from light sources, which can compromise sensitivity. Combined with single-photon counting detectors, these systems can achieve the ultimate detection limits, even in the single-molecule range. For example, chemiluminescent assays based on AMPPD (see chemical structure in Figure 1) have demonstrated detection limits in the attomole region, highlighting the exceptional sensitivity achievable with such techniques (Figure 7) [61,63].

Shortly after their discovery, chemiluminescent 2-coumaranones were explored for similar bioanalytical applications. Over the years, assays targeting various substrates and enzymes, including peroxidase, glucose, and urease, have been developed by multiple researchers, showcasing the versatility of these compounds in bioanalytical contexts [43,49,50,54,56,59]. Beyond bioanalysis, chemiluminescent 2-coumaranones have found applications in material science, particularly as mechano-base-sensing probes. This innovative use leverages the ability of 2-coumaranones to emit light under mechanical stress, opening new possibilities for stress and strain sensing in materials [53].

Another novel application involves the use of 2-coumaranones as chemiluminescent protective groups. These protective groups have been studied in-depth by Lippold et al., who explored their synthesis and utility in photochemical applications. They demonstrated their potential in controlled light-emitting reactions, adding to the functionality of chemiluminescent systems in advanced chemical and material applications [56], further solidifying 2-coumaranones role as indispensable tools in chemiluminescence research and applications.

### 6.1. Aqueous Chemiluminescence of 2-Coumaranones Using Peroxidase/H_2_O_2_

Although the base-induced chemiluminescence of 2-coumaranones is generally limited to aprotic polar solvents, such as acetonitrile, acetone, or DMF, Matuszczak’s 1991 thesis revealed a surprising alternative mechanism. She demonstrated that the chemiluminescence of 2-coumaranones can also be triggered in aqueous environments through the addition of hydrogen peroxide (H_2_O_2_) and the enzyme peroxidase in phosphate buffer with EDTA [19]. In these experiments, the 2-coumaranones were pre-dissolved in DMSO or acetonitrile, but the bulk of the reaction volume consisted of water. This suggests that bioanalytical applications of the chemiluminescence measurement could be feasible in aqueous systems.

Matuszczak further observed that the chemiluminescent activity of the H_2_O_2_/peroxidase-triggered reaction is pH-dependent, reaching a plateau above pH 8–9. Additionally, the luminescence intensity was proportional to the H_2_O_2_; concentration, indicating that H_2_O_2_ functions as the primary oxygen source in this mechanism, as opposed to triplet oxygen (O_2_) used in base-induced chemiluminescence [41]. This highlights a fundamental difference between the two reaction pathways.

**Figure 8:**
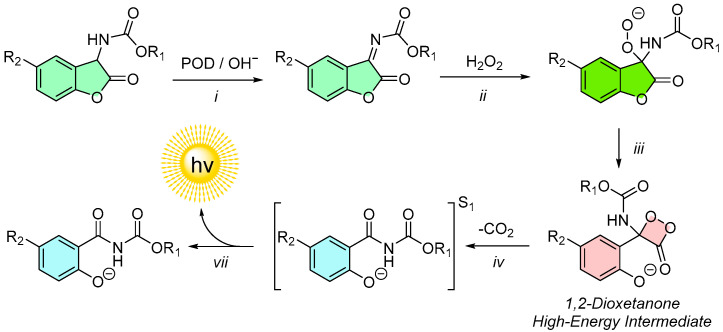
Hypothetical mechanism of the peroxidase (POD)/H_2_O_2_ initiated chemiluminescence of 2-coumaranones.

To explain this behavior, Matuszczak proposed a reaction mechanism (Figure 8) [41]. The mechanism involves the following steps: Dehydrogenation: The 2-coumaranone molecule undergoes enzymatic dehydrogenation by peroxidase, forming an N-acylimine intermediate (i). This reaction aligns with peroxidase’s known ability to dehydrogenate various organic molecules [64]. Peroxide Attack: The N-acylimine is subsequently attacked by H_2_O_2_, leading to the formation of a peroxy-anion intermediate (ii). Cyclization: The peroxy-anion cyclizes into a 1,2-dioxetanone (iii), which acts as the high-energy intermediate of the reaction. Decarboxylation and Light Emission: The 1,2-dioxetanone decarboxylates, generating salicylamide-like emitters in an excited state (iv). These emitters release energy in the form of light as they transition back to the ground state.

This mechanism not only explains the aqueous chemiluminescence of 2-coumaranones but also opens pathways for practical applications. For instance, it enables the development of analytical assays for H_2_O_2_ and peroxidase detection, similar to those based on luminol. The measurement principle was further elaborated by Matuszczak in her 1996 publication, where she described assays utilizing this chemiluminescence system [43].

Building on this foundation, a logical next step is the construction of luminescence-based immunoassays. Such assays could involve antibodies conjugated to peroxidase, where chemiluminescence is triggered only upon antibody binding to a specific target. This binding would localize the peroxidase and catalyze the chemiluminescence reaction, providing a highly sensitive and specific method for target detection.

### 6.2. Glucose Detection Using 2-Coumaranone Chemiluminescence

Matuszczak demonstrated that the chemiluminescence reaction of 2-coumaranones, catalyzed by peroxidase (POD) and hydrogen peroxide (H_2_O_2_), can be coupled with glucose oxidase (GOD) to enable the determination of glucose concentrations in aqueous solutions [41,43]. This enzymatic coupling allows the indirect quantification of glucose by linking its oxidation to the chemiluminescence of 2-coumaranones.

**Figure 9:**
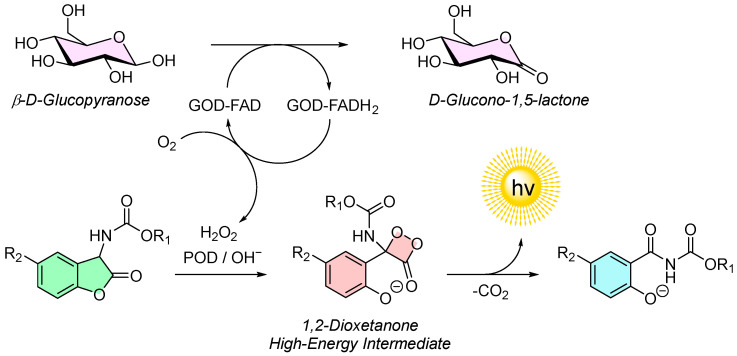
Glucose assay utilizing the peroxidase (POD)/H_2_O_2_ initiated chemiluminescence of 2-coumaranones coupled to the oxidation of glucose to gluconolactone by glucose oxidase flavin adenine dinucleotide (GOD-FAD) complex.

In this system, glucose is first oxidized by the GOD-flavin adenine dinucleotide (GOD-FAD) enzyme complex, resulting in the production of gluconolactone and the reduced enzyme complex GOD-FADH_2_. The GOD-FADH_2_ complex then reacts with molecular oxygen, regenerating GOD-FAD and producing H_2_O_2_ as a by-product. The generated H_2_O_2_ subsequently acts as a reagent in the POD-catalyzed chemiluminescence reaction of 2-coumaranones, as described earlier (Figure 9). The light output from this reaction correlates directly with the initial glucose concentration, allowing for quantitative analysis.

Matuszczak reported that this assay exhibits excellent linearity up to glucose concentrations of 400 mg/100 mL, encompassing both physiological and pathological blood-glucose ranges (80–140 mg/100 mL) [41,43,65]. This broad linear range underscores the assay’s potential for both clinical and laboratory applications.

The further development of such chemiluminescence-based assays, particularly their integration into modern point-of-care diagnostic devices, could significantly advance the field of blood-glucose monitoring. This approach offers the prospect of highly sensitive, easy-to-use diagnostic tools for managing diabetes and other conditions that affect glucose metabolism.

Beyond glucose detection, the underlying principle of enzymatic substrate oxidation can likely be extended to other important biomolecules. For example, the oxidation of cholesterol by cholesterol-oxidase could serve as the basis for a similar chemiluminescent assay, enabling the quantification of cholesterol in biological samples. This adaptability suggests that chemiluminescence-based assays using 2-coumaranones have the potential to be generalized for a wide range of biomolecules, paving the way for new analytical methods in biochemical and clinical diagnostics.

### 6.3. Urease-Triggered Chemiluminescence of 2-Coumaranones

Urease is an enzyme that catalyzes the hydrolysis of urea into carbon dioxide and ammonia. It is widely distributed across bacteria, fungi, algae, and plants, where it plays critical biological roles. Medically, urease is implicated in the pathogenesis of various conditions, including *Helicobacter pylori* infection, hepatic encephalopathy, hepatic coma, and infection stones [44]. In agriculture, urease activity leads to the rapid decomposition of urea-based fertilizers, contributing to environmental damage such as nitrogen loss and pollution [66].

In 2016, Schramm first reported preliminary findings on the chemiluminescence activity of carbamate-type 2-coumaranones upon the addition of urease. The chemiluminescence was hypothesized to result from the hydrolysis of the carbamate moiety, yielding an amine whose basicity triggers the chemiluminescence of 2-coumaranones [50]. Building on this concept, Krieg et al. synthesized a wide variety of 2-coumaranones featuring urea subunits instead of carbamates. Although these derivatives were expected to undergo easier hydrolysis by urease, the authors did not report on their chemiluminescence activity with the enzyme [54].

A more detailed investigation into the interaction between 2-coumaranones and urease was undertaken by Baranov et al. in 2024 [59]. They synthesized a range of 2-coumaranones containing either carbamate or urea subunits and systematically evaluated their chemiluminescence activity under various conditions, including the addition of bases in polar aprotic solvents, H_2_O_2_ in water, and urease in aqueous media. Their findings highlighted that certain urea-based 2-coumaranones, particularly those with small urea moieties and *para*-position substituents ranging from slightly deactivating to strongly activating, exhibited medium to strong chemiluminescence activity in the presence of urease [59].

**Figure 10:**
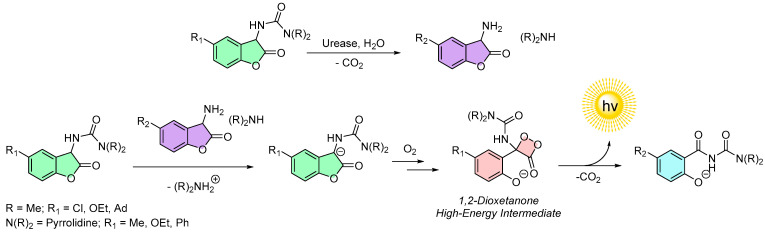
Urease triggered chemiluminescence of 2-coumaranones bearing urea moiety and derivatives that shows this reaction with medium to strong chemiluminescence activity.

Baranov et al. adapted the earlier mechanism proposed by Schramm to explain this phenomenon (Figure 10). In this mechanism, urease catalyzes the hydrolysis of the urea subunit in the 2-coumaranone, producing carbon dioxide, alpha-amino-2-coumaranone, and an aliphatic amine [59]. Both the alpha-amino-2-coumaranone and the aliphatic amine exhibit sufficient basicity to deprotonate unreacted 2-coumaranone; however, the stronger basicity of the aliphatic amine predominates. This results in the formation of the deprotonated 2-coumaranone and an ammonium salt. The deprotonated 2-coumaranone then undergoes the same base-induced chemiluminescence mechanism as previously described, involving the formation of a high-energy 1,2-dioxetanone intermediate and a salicylamide-like emitter structure, culminating in light emission.

While these findings are promising, the intensity of the urease-triggered chemiluminescence was found to be at least one order of magnitude lower than the chemiluminescence observed in DBU-induced reactions in polar aprotic solvents [59]. Consequently, further research is required to enhance the luminescence intensity of urease-triggered chemiluminescence reactions before this system can be developed into practical analytical tools, such as chemiluminescent tests for pathogens like *Helicobacter pylori*.

### 6.4. Mechano-Base-Sensing with 2-Coumaranone Chemiluminescence

Over recent decades, advances in the study of mechanical phenomena, particularly in soft materials and biological systems, have spurred interest in mechanoresponsive materials. These materials are used to study and quantify mechanical actions such as stress and strain in polymers and crystalline systems [67,68,69,70,71,72]. Among these, mechanoluminescence has emerged as a promising approach for probing mechanical forces, with Sijbesma et al. extending this concept to chemiluminescence through mechano-base-induced mechanisms [53].

To investigate this, Sijbesma’s group analyzed the base-induced chemiluminescence of dioxetanes and 2-coumaranones. A critical step in their approach involved finding a method to mechanically induce the production of a base capable of triggering the chemiluminescence reaction. For this purpose, they utilized a coordination polymer of poly(tetrahydrofuran) centrally functionalized with N-heterocyclic carbene (NHC) palladium complexes. These complexes are highly susceptible to mechanochemical scission, such as when exposed to ultrasound. Upon mechanical cleavage, the released NHC acts as a base to initiate the chemiluminescence reaction.

The chemiluminescence mechanism of this mechano-base-induced reaction is presumed to mirror the conventional base-induced chemiluminescence of 2-coumaranones. In both cases, the base deprotonates the 2-coumaranone, leading to the formation of a high-energy 1,2-dioxetanone intermediate which undergoes decarboxylation, resulting in an excited state emitter species that gives off light as it transitions back to the ground state.

Sijbesma’s group successfully demonstrated the coupling of mechano-base release from the palladium-NHC coordination polymer with the chemiluminescence of 2-coumaranones in toluene as the solvent. However, they did not show similar chemiluminescence reactions in the pure solid state, such as in polymer matrices or co-crystals. This limitation suggests that the reaction currently requires a liquid medium to function effectively.

Despite this limitation, the potential of mechano-base-induced chemiluminescence is significant. It could serve as a novel method for generating chemiluminescence through mechanical stimuli, offering applications in stress sensing and as a probe for bond scission processes in materials. Future research may focus on adapting this system for use in pure solid-state environments, which would greatly expand its applicability in mechanoresponsive materials and devices.

### 6.5. Chemiluminescent Protection Group

In total synthesis of complex organic molecules, protecting and cleaving functional groups are essential to prevent side reactions. Recently, photoprotecting groups (PPGs), engineered as chromatically orthogonal protective groups, have emerged as remarkable tools. These systems utilize light—either through direct absorption or photocatalysis—to enable efficient and non-invasive deprotection [73].

A complementary approach involves protecting groups whose cleavage is accompanied by photon emission, termed chemiluminescent protecting groups (CLPGs). The key advantage of CLPGs is their ability to facilitate in situ monitoring of synthesis steps, as the visible chemiluminescence confirms successful and complete deprotection, eliminating the need for additional analytical verification.

**Figure 11:**
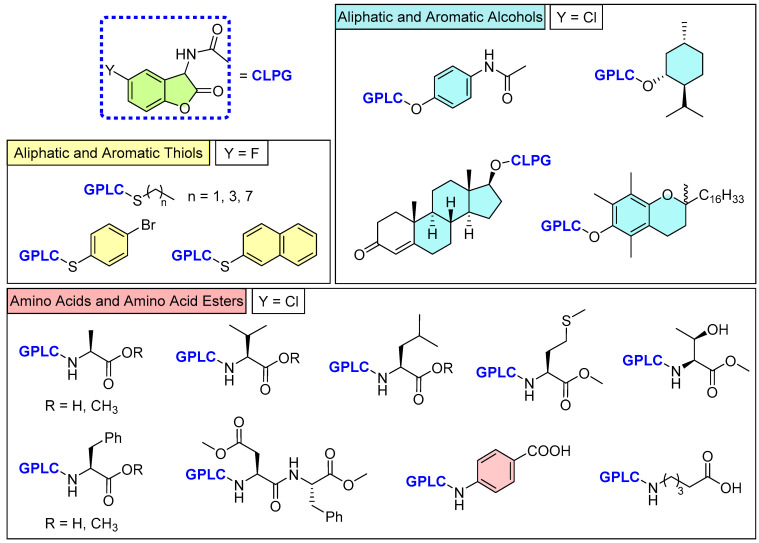
Overview of synthesized compounds by Lippold [56,57] for each substrate class (amines, alcohols, and thiols) with the respective yields.

For effective application, CLPGs must meet specific criteria: (1) they should exhibit bright chemiluminescence within the visible spectrum that ceases upon deprotection, and (2) they must not display interfering absorption or fluorescence before activation.

(De)protection of aliphatic and aromatic amines (focusing on (non-)proteinogenic amino acids), alcohols, and thiols using 2-coumaranones has been studied by Lippold et al. (Figure 11) [56,57]. The findings demonstrate that the effectiveness of the CLPG concept is highly dependent on the charge stabilization of the potential nucleofuge and the heteroatom attached to the amide side chain of the benzofuranone.

None of the urea-2-coumaranone derivatives studied were capable of releasing the corresponding amino acids, likely due to the poor leaving group potential of these compounds. The observed weak and transient chemiluminescence can be attributed to the preferential nucleophilic attack of the urea moiety on the critical 1,2-dioxetanone intermediate, which results in the formation of either a hydantoin or an open-chain carboxylate species, with the equilibrium between these two structures being derivative-dependent. Luminescence experiments further suggested that the analyzed urea-2-coumaranones produced a similar fluorescent species, as indicated by their comparable absorption and emission spectra. This fluorescence is believed to originate from the open-chain derivative. Notably, amino acid esters displayed delayed photoluminescence compared to their unprotected counterparts, which fluoresced immediately upon DBU addition. This indicates that both the protection of the carboxylic acid and the amino acid side chain significantly influence the equilibrium involved in forming the fluorescent species. Upon acidification, the equilibrium shifted entirely towards the hydantoin structure, which could then be isolated. However, a different outcome emerged after hydrolyzing the oxidized urea-2-coumaranone derivative of PABA. In this case, the main product was the urea-protected PABA compound, with completely deprotected PABA appearing as a by-product. The direct attachment of the amine group to an aromatic ring appears to facilitate further decomposition in mildly acidic aqueous conditions, likely due to enhanced charge stabilization (Figure 11).

Due to these results, the urea-2-coumaranones were termed as fluorescent protecting groups (FPGs), as they do not fulfil the requirements of a CLPG, but could be considered for their potential as a visualizing tool for biologically relevant amine compounds and enabling medical imaging applications.

Contrary to amines, aromatic alcohols and thiols are released quickly, accompanied by a shorter chemiluminescence duration due to their remarkable leaving group potential and a higher probability of a dark side reaction. Aliphatic derivatives are only completely cleaved from the CLPG in the case of thiols.

With these results it can be stated that 2-coumaranones represent a fascinating PG alternative, specifically for phenols and thiols, which can be removed either in alkaline solutions with non-nucleophilic bases or enzymatically (e.g., with peroxidase). Apart from the leaving group potential, minimal steric hindrance is of great importance also, further specifying the demands of substrates that sought to be protected. With the emphasis on biological and medical applications, the release of 2-coumaranone-protected molecules could open up new methods, e.g., for imaging techniques.

## 7. Conclusions, Open Questions and Future Directions

In summary, the study of chemiluminescent 2-coumaranones has evolved from an intriguing chemical curiosity to a robust and versatile platform with broad applicability. Over 110 derivatives have been reported—many accessible via the convenient “One-Pot” Tscherniac–Einhorn 3-component reaction, with some even reaching commercial availability [74]. This facile synthesis, coupled with the remarkable tunability of their photophysical properties, has positioned 2-coumaranones at the forefront of chemiluminescence research.

Most derivatives emit deep blue light (~420–450 nm), but by extending the π-conjugated system or modifying substituents on the aromatic core, emission can be shifted to green or orange (up to 580 nm). Moreover, the reaction kinetics can be finely tuned—ranging from flash-like to glow-type emissions—by strategic alterations of steric factors. These properties already rival those of established systems like luminol, especially given that quantum yields as high as ~8% have been achieved in select derivatives.

Extensive mechanistic studies have delineated the pathway involving deprotonation, formation of a high-energy 1,2-dioxetanone intermediate, and subsequent generation of a deprotonated salicylamide-like emitter. Despite these advances, some side reactions—likely involving alternative dark pathways—remain hypothetical. A deeper understanding of these competing processes, particularly the interplay of steric and electronic effects, could enable the rational design of even more efficient systems.

Similarly, the accidental discovery of 2-coumaranone-based electrochemiluminescence when interfaced with platinum electrodes hints at entirely new reaction channels that remain to be elucidated [49,50].

The versatility of 2-coumaranones has been showcased in a variety of applications—from enzyme-catalyzed assays (glucose detection, urease-triggered reactions) and mechano-base-sensing to innovative chemiluminescent protecting groups. Their potential to serve as superior alternatives to traditional luminol-based systems, due to higher quantum yields and tunable spectral and kinetic properties, is particularly promising.

Nevertheless, there are still many as yet unanswered questions that open up great potential for future research directions:

**(1) Expanding the Spectral Range:** Although the emission color of 2-coumaranones can be tuned from blue to orange (420–580 nm), derivatives that emit in the deep red or near-infrared (NIR) regions are still lacking. Achieving these wavelengths is crucial for applications such as deep-tissue bioimaging, where minimal light scattering and tissue absorption are required.

**(2) Mechanistic Elucidation of Side Reactions:** While the primary chemiluminescence pathway is well characterized, several side reactions remain speculative. In particular, understanding the factors that suppress the dark reaction channels—such as sterically demanding substituents—could further enhance quantum yields. Detailed studies into the electronic and steric influences on these competing pathways are needed.

**(3) Deciphering Electrochemiluminescence Mechanisms:** The serendipitous observation of 2-coumaranone-based electrochemiluminescence by Schramm opens an entirely new dimension for research. Understanding the underlying mechanism could lead to the development of tailored electrochemiluminescent assays and sensors [49,50].

**(4) Broadening Application Horizons:** The potential applications for 2-coumaranones are vast and still largely unexplored. Future investigations might focus on:

**(5) Chemiluminescent Immunoassays (ELISAs):** Developing robust, high-sensitivity assays by replacing conventional substrates with 2-coumaranones.

**(6) Ultrasensitive Biosensing:** Utilizing these compounds for oxygen sensing, ATP detection, and monitoring reactive oxygen species.

**(7) Expanding Enzymatic Assays:** Extending the scope of oxidation-based assays (e.g., for cholesterol or other biomolecules) by leveraging the high quantum yields of these systems.

**(8) Point-of-Care Diagnostics:** Exploiting the tunability and efficiency of 2-coumaranones for rapid, on-site diagnostic tests (e.g., urease activity in *Helicobacter pylori* infections).

Chemiluminescent 2-coumaranones have established themselves as a versatile and highly tunable class of organic chemiluminophores. Their ease of synthesis, impressive quantum yields, and adaptable emission characteristics render them exceptionally promising for both fundamental studies and practical applications. However, critical challenges remain—chief among them, the expansion of the emission spectrum into the red/NIR region, a deeper mechanistic understanding of side and novel reaction pathways, and the translation of these findings into real-world analytical and diagnostic tools.

By addressing these open questions, future research will not only enhance our fundamental understanding of chemiluminescence but also drive the development of next-generation chemiluminescent technologies with unprecedented sensitivity, selectivity, and application versatility.

## Supplementary Materials

The following supporting information can be downloaded at: https://www.mdpi.com/article/10.3390/molecules30071459/s1, Table S1: Overview on photophysical properties of selected 2-coumaranones; Figure S1: Examples for Chemiluminescence, Fluorescence after Chemiluminescence decay and Absorbance spectra of 2-coumaranones.
