# Supplementary material for: Chemiluminescent 2-Coumaranones: Synthesis, Luminescence Mechanism, and Emerging Applications
Source: Molecules. 2025 Mar 25;30(7):1459. doi: 10.3390/molecules30071459 (PMC11990580; doi:10.3390/molecules30071459)
Supplement: Supplementary file 1 [file molecules-30-01459-s001.zip › molecules-3523615-supplementary.pdf]

**Table S1:** Overview on selected chemiluminescence, fluorescence after chemiluminescence decay and absorbance properties of 2-Coumaranones

| Structure | Chemiluminescence Maximum [nm] | Fluorescence Maximum [nm] | Absorbance Maximum [nm] | Reference |
|---|---|---|---|---|
| 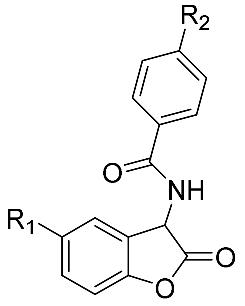 $R_1$ = Br, $R_2$ = H | 514 | 486 | NA | [1] |
| 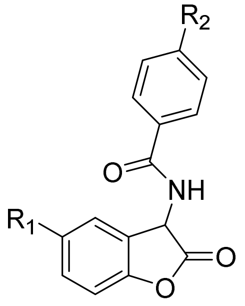 $R_1$ = Cl, $R_2$ = H | 512 | 494 | NA | [1] |
| 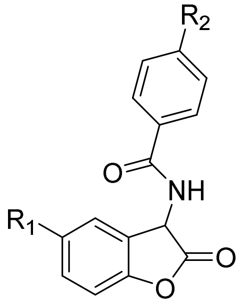 $R_1$ = F, $R_2$ = H | 490 | 480 | NA | [1] |
| 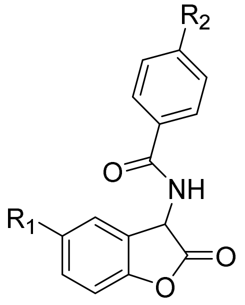 $R_1$ = Br, $R_2$ = $CH_3$ | 498 | 500 | NA | [1] |

| | | | | |
|---|---|---|---|---|
| 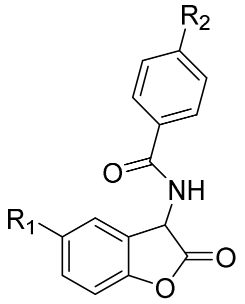 $R_1$ = Br, $R_2$ = Br | 562 | 559 | NA | [1] |
| 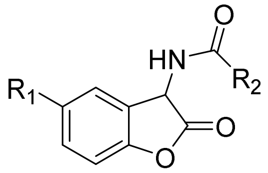 $R_1$ = Br, $R_2$ = $(CH2)_{12}CH_3$ | 452 | 458 | NA | [1] |
| 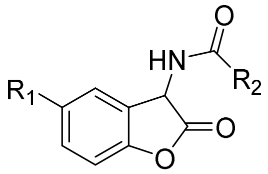 $R_1$ = Br, $R_2$ = O-Butyl | 448 | 450 | NA | [1] |
| 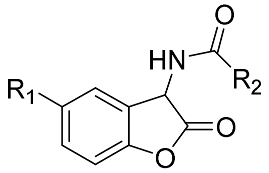 $R_1$ = F, $R_2$ = O-Et | 433 | 434 | ~280, 340, 380 | [2,3] |
| 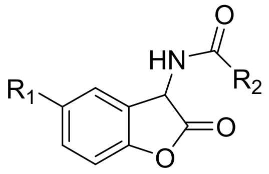 $R_1$ = F, $R_2$ = O-Me | 436 | 437 | NA | [3] |
| 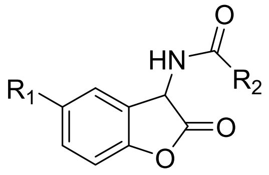 $R_1$ = Cl, $R_2$ = O-Me | 427 | 428 | NA | [3] |
| 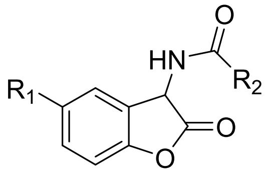 $R_1$ = Br, $R_2$ = O-Me | 427 | 428 | NA | [3] |

| | | | | |
|---|---|---|---|---|
| 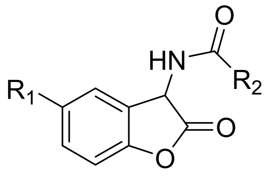 $R_1$ = Cl, $R_2$ = O-Et | 427 | 428 | NA | [3] |
| 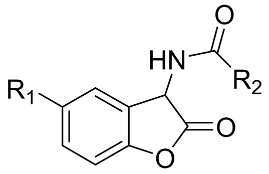 $R_1$ = Br, $R_2$ = O-Et | 428 | 426 | NA | [3] |
| 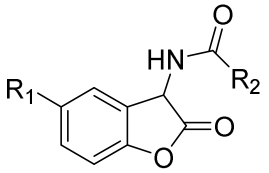 $R_1$ = F, $R_2$ = O-$CH_2C(CH_3)_3$ | 435 | 436 | NA | [3] |
| 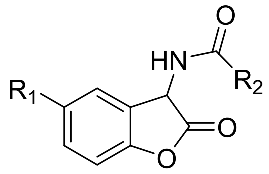 $R_1$ = F, $R_2$ = O-Propargyl | 437 | 435 | NA | [3] |
| 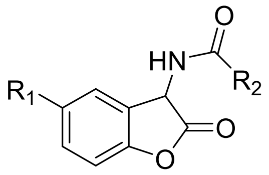 $R_1$ = F, $R_2$ = O-$(CH_2)_6N_3$ | 434 | 433 | NA | [3] |
| 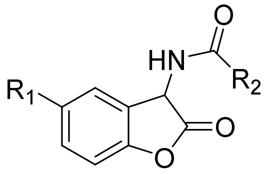 $R_1$ = F, $R_2$ = O-$C_6H_4$-4-F | 445 | 446 | NA | [3] |
| 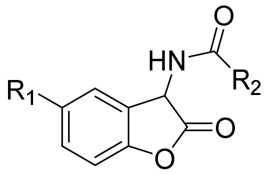 $R_1$ = F, $R_2$ = O-$C_6H_4$-2-Br | 432 | 438 | NA | [3] |
| 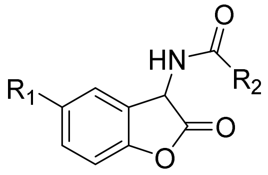 $R_1$ = F, $R_2$ = O-Mesityl | 442 | 444 | NA | [3] |

| | | | | |
|---|---|---|---|---|
| | 444 | 408 | NA | [3] |
| | 448 | 438 | NA | [3] |
| | 472 | 478 | NA | [3] |

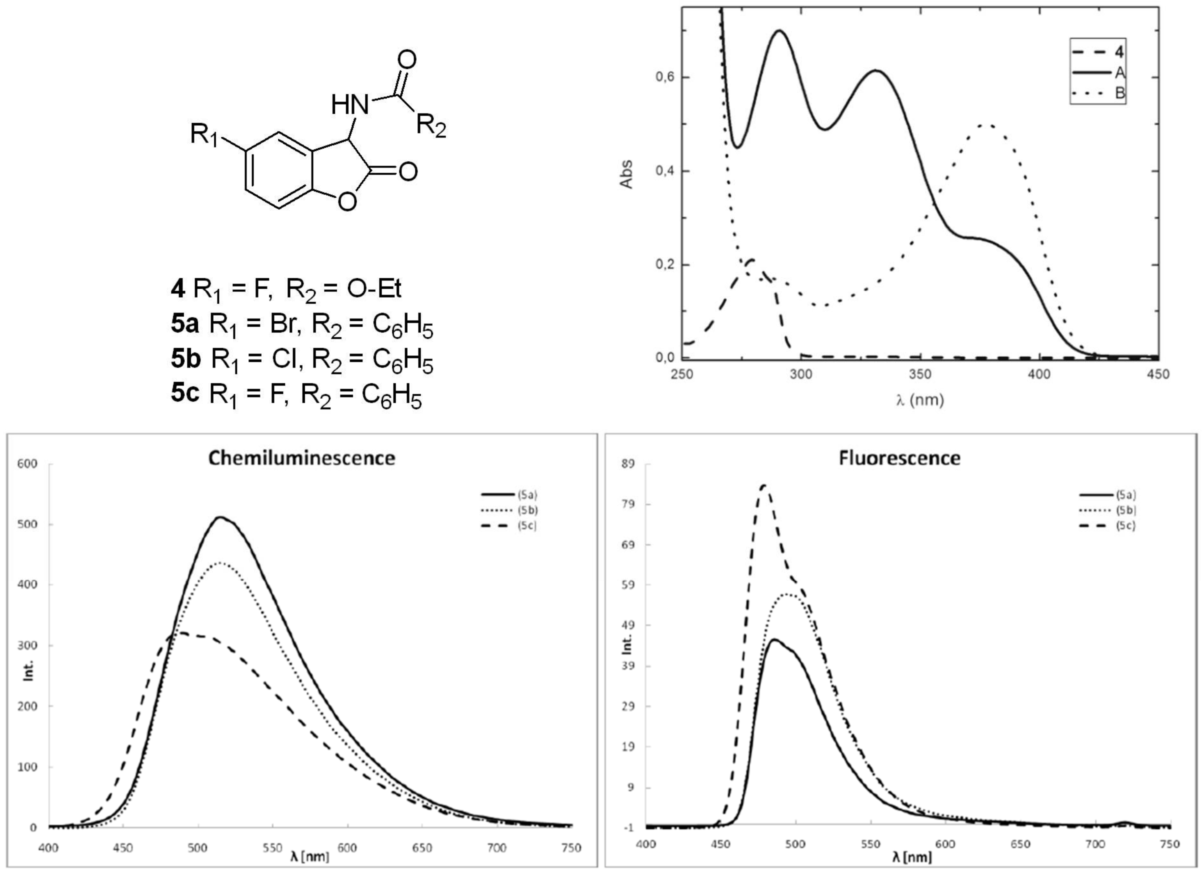


**Figure S1:** Examples for Chemiluminescence, Fluorescence after Chemiluminescence decay and Absorbance spectra from Reference [1-3].

## SI-References


1. Schramm, S.; Weiss, D.; Navizet, I.; Roca-Sanjuán, D.; Brandl, H.; Beckert, R.; Görls, H. Investigations on the synthesis and chemiluminescence of novel 2-coumaranones. *ARKIVOC* **2013**, 174 - 188.
2. Ciscato, L.F.M.L.; Bartoloni, F.H.; Colavite, A.S.; Weiss, D.; Beckert, R.; Schramm, S. Evidence supporting a 1,2-dioxetanone as an intermediate in the benzofuran-2(3H)-one chemiluminescence. *Photochemical & Photobiological Sciences* **2014**, *13*, 32-37, doi:10.1039/c3pp50345c.
3. Schramm, S.; Ciscato, L.F.M.L.; Oesau, P.; Krieg, R.; Richter, J.F.; Navizet, I.; Roca-Sanjuán, D.; Weiss, D.; Beckert, R. Investigations on the synthesis and chemiluminescence of novel 2-coumaranones - II. *ARKIVOC* **2015**, *2015*, 44-59, doi:10.3998/ark.5550190.p009.044.
